# The Use of the File

**Published:** 1865-02

**Authors:** S. Trowbridge


### THE USE OF THE FILE.

Read before the Iowa State Dental Society, January 4th, 1865.

BY S. TROWBRIDGE.

The file should be used for two purposes in dental operations.

1st. To remove superficial decay.

2nd. As a preparatory step to filling where the decay has progressed so far that it is not advisable to remove all of the decay by the use of the file.

The cases, where superficial decay is most frequently removed, are on the central and lateral incisors and canines.

For this purpose a flat safe-sided finely cut file is most convenient. The edges of the file for removing decay on the approximal surfaces of the teeth should be beveled so as effectually to prevent any shoulder being formed on the tooth near the margin of the gums, and here we remark that for theoretical, and we believe for practical utility, the handles of these, as well as all separating files, are placed on the wrong end of the file; that is, the files are made to cut pushing from instead of drawing toward the operator, whereas, they should be made to cut toward the operator and not from him.

The advantage of this will readily be seen from the construction of the dental arch, which is such that a pressure sufficient to cut off a portion of the enamel will tend to diminish the circle of which this is an arc, and the circle thus diminished, the file is grasped firmly between the teeth, causing a disagreeable, if not injurious concussion.

But were the pressure made so as to enlarge the circle and increase the space between the teeth, the operation could be performed with less liability to injure the teeth, and more agreeably to the patient. So much for the file itself, now for the results of its use.

Does it injure the teeth to file them? We answer, yes, It injures them inasmuch as it takes off a part of the covering or enamel which nature has placed there for protection of the more vital parts of the tooth, and as disease in teeth as well as in the general system, finds its first hold in the weakest points, we have, seemingly, given it an encouraging commencing place. But, as in the first case, we would use the file only for the removal of actual decay, it becomes a question, not whether filing sound teeth injures them, but whether the removal of the decayed and diseased portion of the tooth, by the use of the file, injures them more than the decay. To this we answer, no, and believe that when thoroughly and properly done, the old adage, "a stitch in time saves nine," is much more appropriate than when applied in its literal signification, and it can be thoroughly and properly done by the use of a fine cut file, and finishing with one that has been worn quite smooth, polishing the surface with a bit of soft wood or flower of emery, or, if between the teeth, with a slip of cloth or chamois skin and flower of emery. But there are very few cases where the dentist is consulted before the case demands a different kind of treatment, and this brings us to the 2d case, where the file should be used, viz:

In preparation of the cavity for filling the file is of great service in separating the teeth, and for the approximal cavities of the six interior teeth a flat safe-sided file is preferable, but for the bicuspids and molars, a V shaped file is better adapted to the work, and unless both teeth are decayed, a safesided file should be used; for it is not allowable to file and destroy the shape of a sound tooth for the purpose of preserving the shape of the diseased one. The opening should be such as to allow the operator to work with certainty of success, and all projecting and irregular points of the enamel on the margins of the cavity should be removed by a small half-round file, and a firm smooth margin left to fill upon and finish.

Neglecting to cut away these thin frail portions of the tooth, for fear of showing the filling, is the cause of failure in nine-tenths of the cases of this kind that fail. This is the groundwork, the foundation, upon which the whole structure rests, and unless this be thoroughly performed, with a thorough knowledge of the ground upon which the foundation is laid, as well as the material used in the superstructure, the whole will indeed be built upon the sand and swept away with complete destruction, to the loss of both patient and operator.

